# Effect of Trimethyltin Chloride on Slow Vacuolar (SV) Channels in Vacuoles from Red Beet (*Beta vulgaris* L.) Taproots

**DOI:** 10.1371/journal.pone.0136346

**Published:** 2015-08-28

**Authors:** Zenon Trela, Zbigniew Burdach, Agnieszka Siemieniuk, Stanisław Przestalski, Waldemar Karcz

**Affiliations:** 1 Department of Physics and Biophysics, Wrocław University of Environmental and Life Sciences, Norwida 25, PL-50-375, Wrocław, Poland; 2 Department of Plant Physiology, Faculty of Biology and Environmental Protection, University of Silesia, Jagiellońska 28, PL-40-032, Katowice, Poland; University of Western Sydney, AUSTRALIA

## Abstract

In the present study, patch-clamp techniques have been used to investigate the effect of trimethyltin chloride (Met_3_SnCl) on the slow vacuolar (SV) channels in vacuoles from red beet (*Beta vulgaris* L.) taproots. Activity of SV channels has been measured in whole-vacuole and cytosolic side-out patch configurations. It was found that addition of trimethyltin chloride to the bath solution suppressed, in a concentration-dependent manner, SV currents in red beet vacuoles. The time constant, τ, increased significantly in the presence of the organotin. When single channel activity was analyzed, only little channel activity could be recorded at 100 μM Met_3_SnCl. Trimethyltin chloride added to the bath medium significantly decreased (by ca. threefold at 100 μM Met_3_SnCl and at 100 mV voltage, as compared to the control medium) the open probability of single channels. Single channel recordings obtained in the presence and absence of trimethyltin chloride showed that the organotin only slightly (by <10%) decreased the unitary conductance of single channels. It was also found that Met_3_SnCl significantly diminished the number of SV channel openings, whereas it did not change the opening times of the channels. Taking into account the above and the fact that under the here applied experimental conditions (pH = 7.5) Met_3_SnCl is a non-dissociated (more lipophilic) compound, we suggest that the suppression of SV currents observed in the presence of the organotin results probably from its hydrophobic properties allowing this compound to translocate near the selectivity filter of the channel.

## Introduction

In the last several decades, organotin compounds have become an important pollutant due to their widespread use in industry and agriculture as fungicides, biocides, antifouling agents, wood preservatives and plastics stabilizers [1–4]. Their high toxicity was established e.g. in algae and aquatic plants [5, 6]. Although their soil concentrations are low and do not usually exceed the micromolar range [7, 8], organotins accumulate in plant tissues and trigger numerous changes in their functioning [9–11]. While inorganic tin is rather unavailable to plants and seems to be hardly toxic, its organic derivatives exhibit much stronger toxicity [12–15]. Despite the large number of papers on organotins, the precise mechanism of their toxic effect on plant cells still remains poorly understood.

Organic compounds of heavy metals, including tin, display significant activity when considering interactions with membranes. According to literature data, the toxic effect of organometallic compounds results from their lipophilic character, facilitating penetration of these radicals through cell membranes and their effect on various cell functions [13, 16, 17].

In order to avoid metal toxicity, plants have evolved two main mechanisms: (1) tolerance to high metal concentrations in cell cytosol and (2) sequestration of metals in vacuoles [18]. The central vacuole, in various plant cell types covering up to 90% of their volume, may be one of the most effective adaptations for deposition and inactivation of heavy metals, helping to reduce their impact on the cytoplasm, being the site of sensitive biochemical processes. Heavy metals may also change the transport systems across the vacuolar membrane (tonoplast), involving channels, carriers and pumps, which play a key role in the control of cytoplasmic homeostasis and cell osmoregulation. Major ion currents at vacuolar membranes of higher plants are mediated by non-selective cation channels: slow-activating vacuolar channels (SV channels) and fast-activating vacuolar channels (FV channels). SV channels were the first channels to be characterized in a vacuolar membrane [19]. They are ubiquitous in every cell type and plant species investigated so far [20–23]. FV channels conduct various monovalent cations with poor selectivity, while SV channels conduct K^+^, Na^+^, Mg^2+^, Ba^2+^, and Ca^2+^ [21]. It has been previously shown that SV channels are regulated by pH [24, 25], calmodulin [24], kinases and phosphatases [26], reducing and oxidizing agents [27], flavonoids [28], 14-3-3 proteins [29], polyunsaturated fatty acids [30], ATP [25], polyamines [31], auxin [32] as well as heavy metals, such as zinc [24], nickel [33, 34] copper [35], and organolead [36, 37].

The main objective of the present study was to determine the effect of trimethyltin chloride on SV channels activity in vacuoles isolated from red beet (*Beta vulgaris* L.) taproots. To the best of our knowledge, no research has been reported on the effects of this organotin compound on SV channels.

## Material and Methods

Vacuoles were isolated from red beet (*Beta vulgaris* L.) taproots with a mechanical method described previously by Coyaud et al. [38]. Surface of a slice of fresh storage tissue was rinsed with bathing solution, enabling the vacuoles to be directly extruded into the recording chamber (1 ml in volume). The control bath solution used in patch-clamp experiments was: 100 mM KCl, 2 mM MgCl_2_, 0.1 mM CaCl_2_, 5 mM MES, 5 mM Tris and 400 mM sorbitol, pH 7.5 adjusted with 0.1 N NaOH, osmolality 650 mOsm. Pipettes were filled with a solution containing: 100 mM KCl, 2 mM MgCl_2_, 1 mM CaCl_2_, 5 mM MES, 5 mM Tris and 340 mM sorbitol, pH 5.5, osmolality 580 mOsm. Osmolality of all solutions was adjusted under the control of cryoscopic osmometer (Semi-Micro Osmometer K-7400, Knauer, Germany). Under symmetrical 100 mM K^+^ and micromolar cytosolic Ca^2+^ concentrations, SV channels conduct outward K^+^ currents (into the vacuole). At Ca^2+^ gradient (0.1 mM in the bath and 1 mM in the pipette) the presence of Ca^2+^ current into the vacuole at depolarizing voltages is also possible. However, it is very difficult to separate K^+^ and Ca^2+^ currents through SV channels because of lack of specific inhibitors of these channels (for review see [22]). All the experiments were performed in two patch-clamp configurations: whole-vacuole and excised cytosolic side-out patch, using the EPC-7 Plus amplifier (List-Medical-Electronic, Darmstadt, Germany), as recently described by Trela et al. [37]. The convention of current and voltage was in accordance with Bertl et al. [39], i.e. the positive (outward) currents indicate an efflux of cations into the vacuole. Experimental data were stored using the Patch-Master software (HEKA Electronic, Lambrecht, Germany). Patch pipettes were pulled from borosilicate glass tubes (Kimax-51, Kimble Products, Toledo, Ohio, USA) using two-stage pipette puller (model L/M-3-PA, List Medical, Germany), fire-polished with a microforge CPZ 101 (List Medical, Germany) and coated with Sylgard (Dow Corning, Midland, MI, USA). Patch electrode resistance (for electrodes filled with pipette solution) was 2–4 MΩ; gigaseal resistance was in the range of 5–20 GΩ. Access to the vacuole interior was gained by breaking the tonoplast under the pipette with a voltage pulse in the range of 300 to 900 mV.

Effects of 1, 10 and 100 μM trimethyltin (Met_3_SnCl) on SV channel activity were studied. Due to the duration of our experiments (the longest lasting 75 min) we decided to use higher tin concentrations than those present in nature. In the experiments, the control bath was changed for a new one of the same salt composition, containing additionally trimethyltin (Met_3_SnCl). The bath solution was exchanged by continuous perfusion of the measuring chamber using an infusion pump (SP200, World Precision Instruments, USA). All experiments were carried out at room temperature (22 ± 1°C). All the data stored were analyzed with FitMaster (HEKA Electronic, Lambrecht, Germany) and computer software Statistica for Windows (StatSoft 2010; STATISTICA data analysis software system, version 9.1., www.statsoft.com, USA). The time course of macroscopic SV currents (whole-vacuole configuration) were fitted with monoexponential function and then two obtained parameters (the steady state current and the time constant of the quasi exponential current) are discussed. In order to compare the results of several experiments, in particular assessing of the effects of trimethyltin, steady state currents were normalized to a value equal to 1 corresponding the steady state current at a membrane voltage of 100 mV in the control bath. In turn, microscopic current traces were used to determine the distribution of the open-close time of the channel and the amplitude of the opening current. Based on current records the opening-closing events characterized by two parameters: duration and amplitude (as average value of the microscopic current during event) were analyzed. Preliminary determination of the levels of the opening-closing currents were carried out by reading the peaks of current histogram (fitted with multimodal gaussian). Individual open-close events are illustrated as a scatterplot. Opening probability was calculated as the total opening time normalized to the total recording time and the number of active channels in patch.

## Results

**Fig 1:**
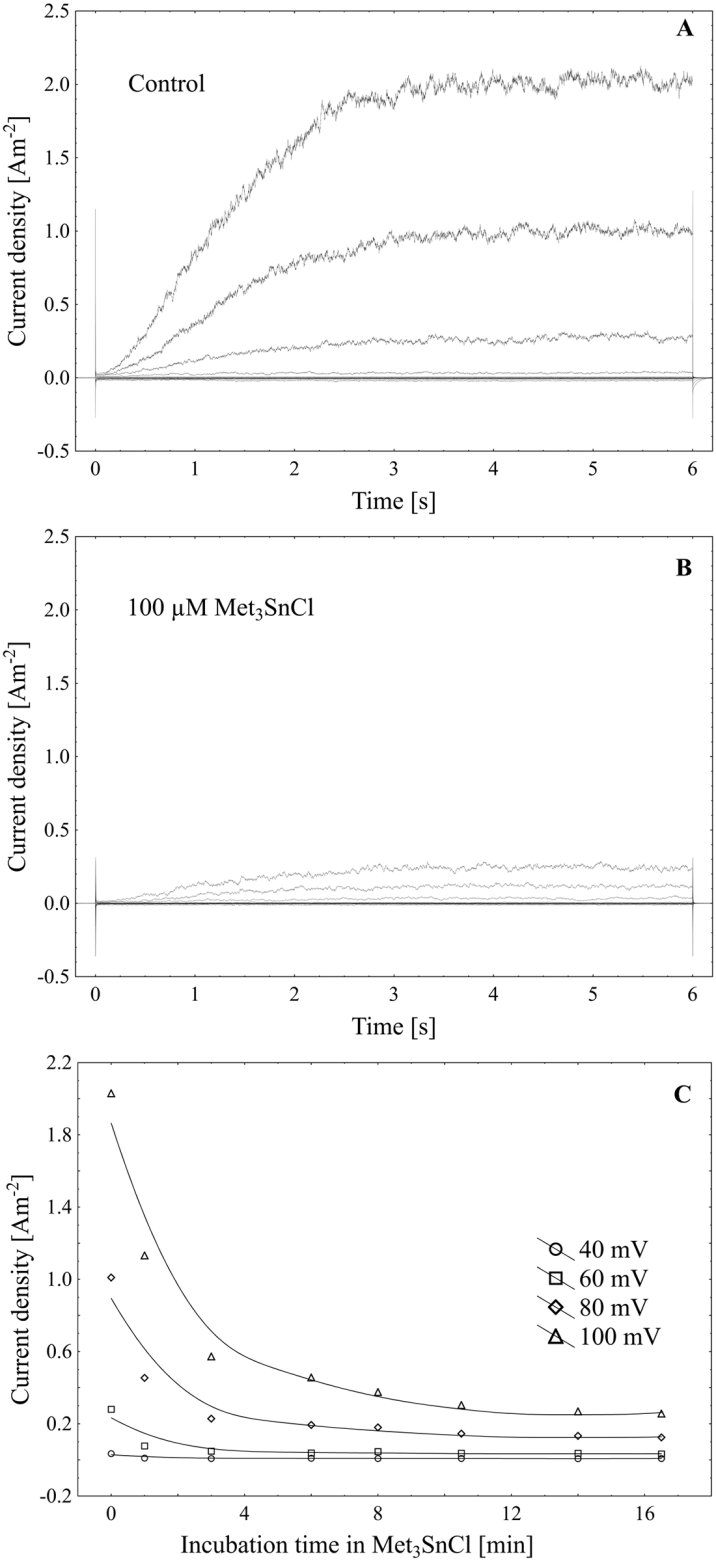
Effect of cytosolic Met_3_SnCl on the slow vacuolar (SV) channels in red beet (*Beta vulgaris* L.) taproot vacuoles. (A) An example of SV current recording for single vacuole in control bath. SV currents elicited by a series of voltage steps ranging from -100 to +100 mV in 20 mV steps; holding potential 0 mV. (B) The same vacuole as in (A) treated with Met_3_SnCl at 100 μM. (C) Steady-state current as a function of time of vacuole incubation in the presence of Met_3_SnCl.

**Fig 2:**
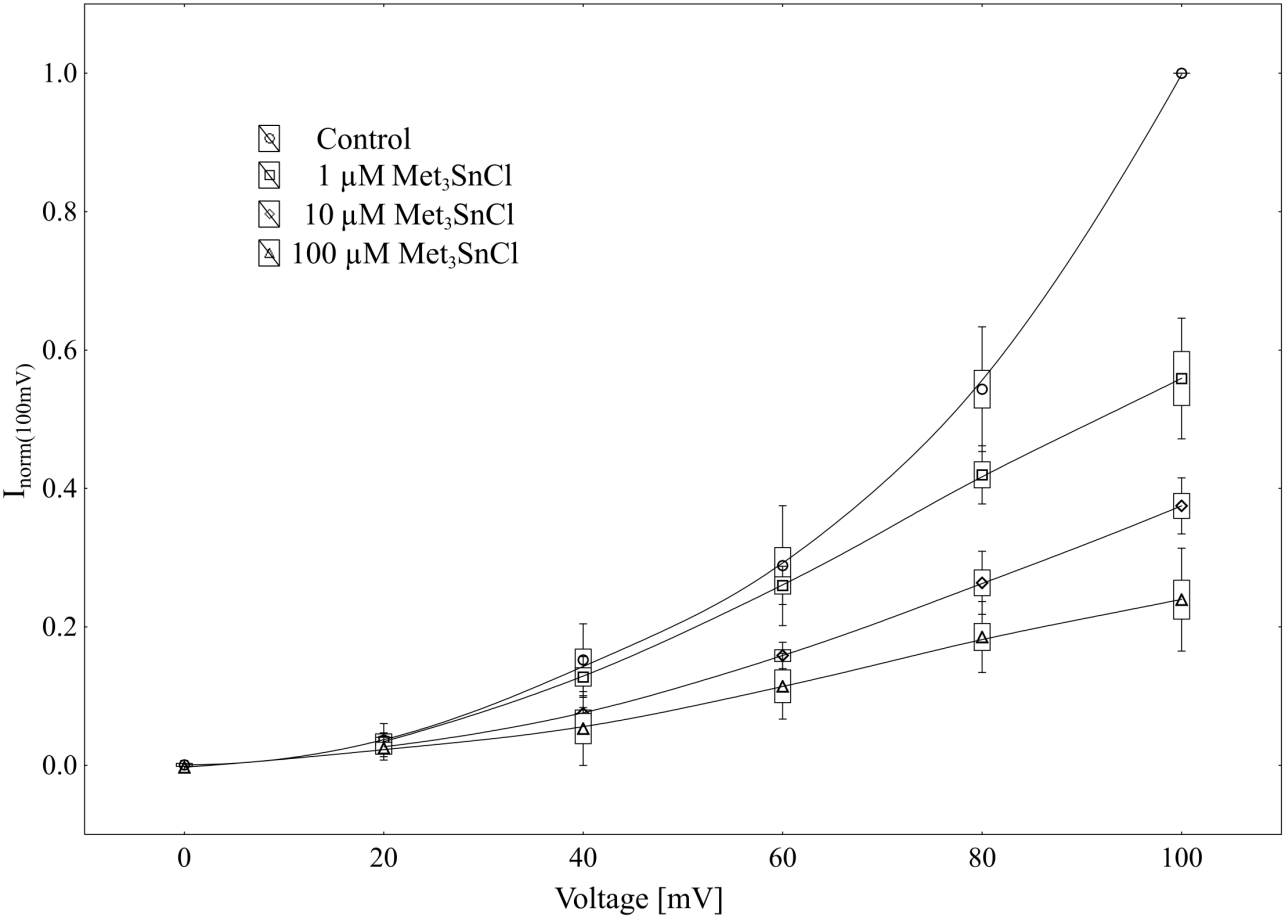
Suppression of macroscopic currents by Met_3_SnCl. Steady-state currents (normalized to the current amplitude determined at +100 mV under control conditions) were determined in the absence or presence of 1, 10 and 100 μM Met_3_SnCl. Data points are means ± SE, SD (n ≥ 6).

**Fig 3:**
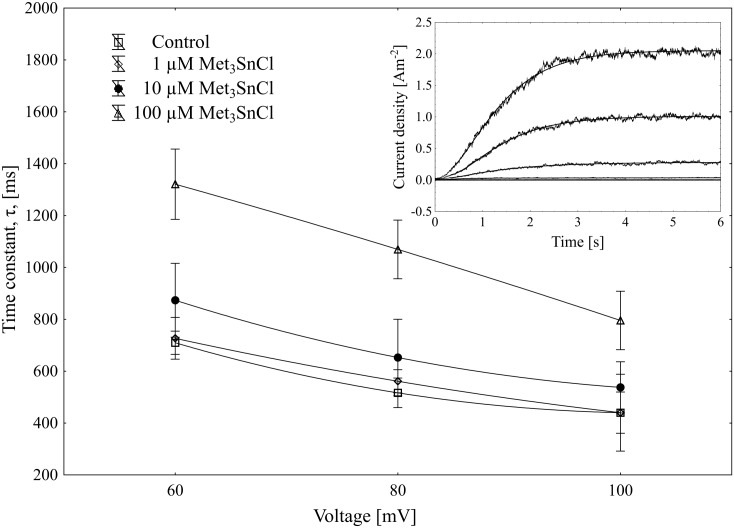
Effect of Met_3_SnCl on activation time, τ, of slow vacuolar (SV) currents. Time constant, τ, as a function of voltage and trimethyltin concentration, shows that addition of Met_3_SnCl (1, 10 and 100 μM) slowed the activation of SV channels. Inset shows an example of macroscopic SV current fitted with a monoexponential function.

**Fig 4:**
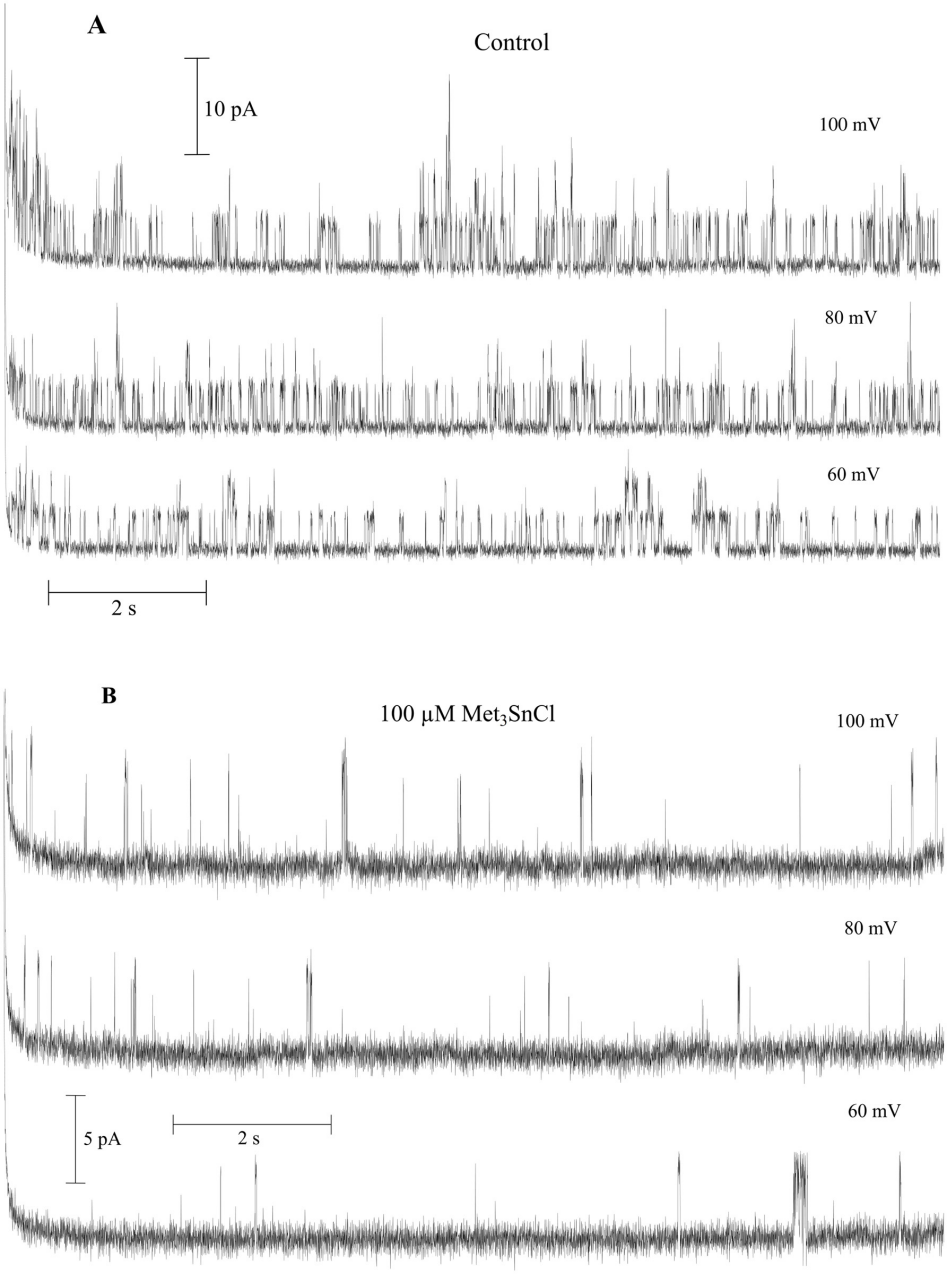
An example of microscopic SV current traces at selected voltages. Single channel openings in control bath (A) and in the presence of 100 μM Met_3_SnCl (B) are shown. The experiments were performed on the same tonoplast patch in the cytosolic side-out configuration. Single channel fluctuations were recorded at +60, +80 and +100 mV.

**Fig 5:**
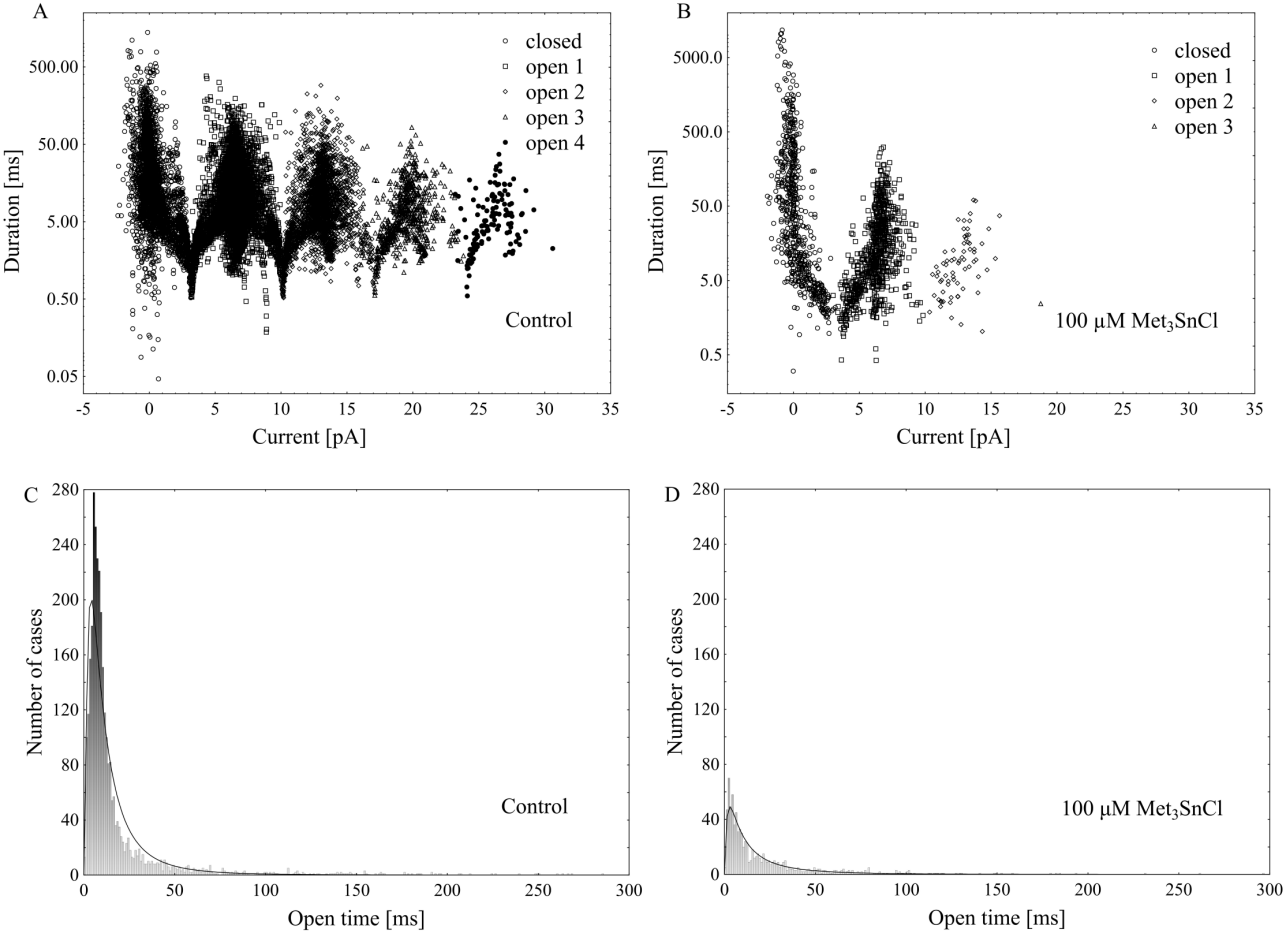
Distribution of times of different current states events (closed, open 1, 2, 3 and 4) as a function of amplitude of current events (i.e. its average value during event) in the control (A) and in the presence of 100 μM Met_3_SnCl (B), and histograms showing the open time of channels under the same conditions, respectively (C and D). In the presence of the organotin the number of opening events was reduced, however the mean time of opening events practically did not change. All events were collected from 10 current traces (each of 12 s duration) obtained at voltage of +100 mV on the same patch, both for the control and in the presence of 100 μM Met_3_SnCl. Histograms showing the times of opening events, fitted with a lognormal distribution function, allowed for obtaining the mean time of opening events and parameter σ, attaining respectively 2.32 ms and 0.97 in the control (C) and 2.46 ms and 1.18 in the presence of 100 μM Met_3_SnCl.

**Fig 6:**
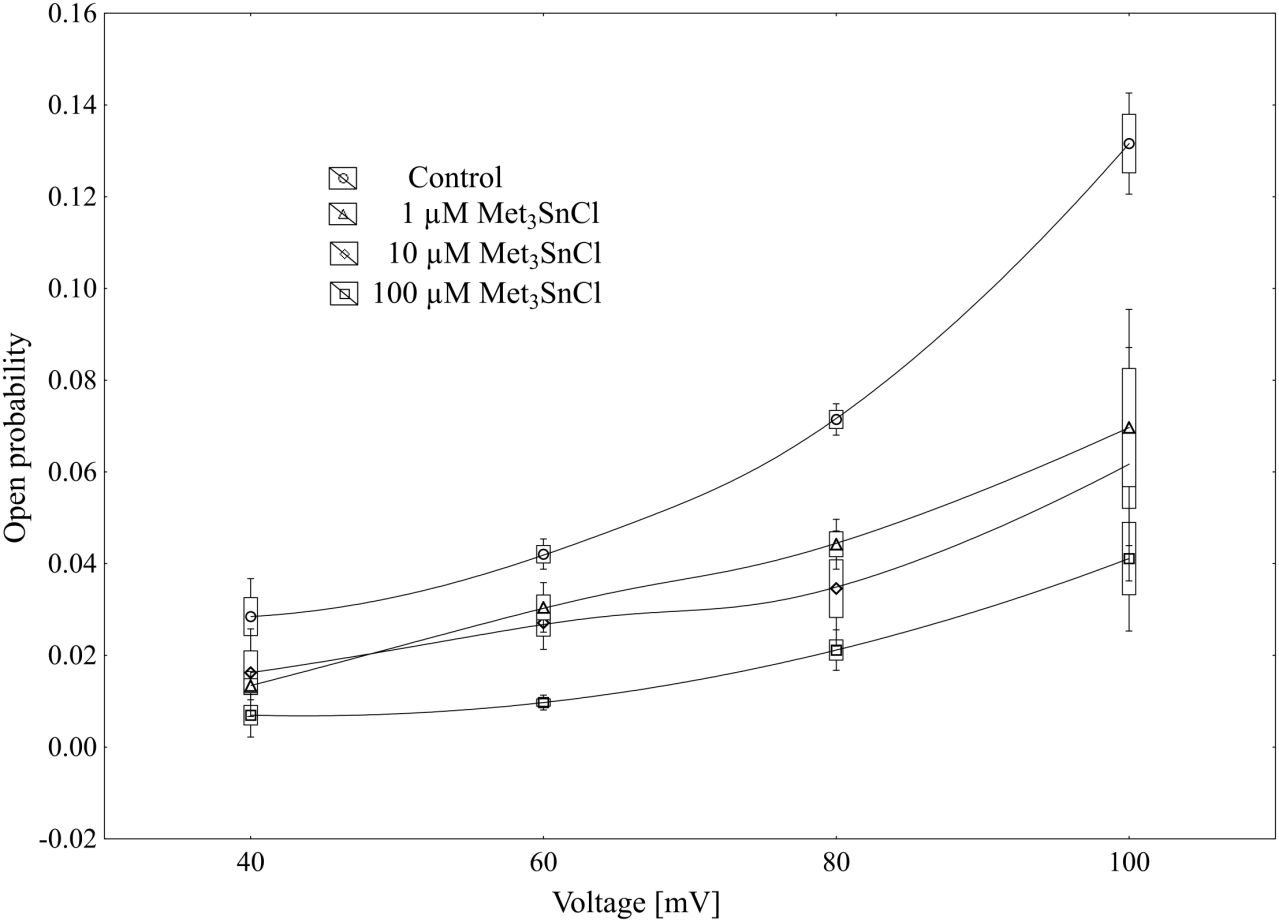
Open probability of slow vacuolar channels as a function of membrane voltage and trimethyltin chloride concentration. Open probability was calculated (using FitMaster software) as a sum of channel open times in the current traces normalized to total time of traces and divided by the number of active channels in the patch. Data points are means ± SE and SD from n ≥ 4 independent experiments.

Using the patch-clamp technique, we examined the effect of trimethyltin chloride (Met_3_SnCl) on SV channel activity in red beet (*Beta vulgaris* L.) taproot vacuoles. Both macroscopic currents (whole-vacuole configuration) and single channel currents (cytosolic side-out configuration) were measured. In the control bath, macroscopic currents showed slow activation typical of steady-state currents (Fig 1A). Addition of Met_3_SnCl to the bath solution, at a final concentration of 100 μM, significantly reduced macroscopic currents (Fig 1B). This effect depended on the time of vacuole incubation in the presence of Met_3_SnCl. As presented in Fig 1C, the greatest changes in macroscopic SV currents, recorded within the first several minutes, were followed by stable and low current values. It is noteworthy that the effect of trimethyltin chloride was irreversible (data not shown). Reduction in macroscopic SV currents progressed with the Met_3_SnCl concentration in bath solution and attained a maximal value at 100 μM (reduction by over 80% at 100 mV, as compared to the control; Fig 2). For example, Met_3_SnCl at 1 μM, applied at the same experimental conditions as 100 μM Met_3_SnCl, diminished SV channel currents by ca. 40% (Fig 2). Fig 3 presents time constants, τ, of monoexponential function fitted to the time courses of macroscopic SV currents. The constants can be interpreted as the rate of SV channel activation after application of voltage pulse. Fig 3 indicates that time constants, τ, increase with organotin concentration, suggesting slower channel activation in the presence of Met_3_SnCl. For example, at 100 μM Met_3_SnCl the time constant, τ, was twofold higher than in the control bath, independently of the applied voltage. When considering microscopic currents in the cytosolic side-out configuration (Fig 4), only little channel activity could be recorded in the presence of 100 μM Met_3_SnCl, compared to the control. This is evident in Fig 5, which shows the distribution of opening-closing events recorded on the same patch of tonoplast both in the control and in the presence of 100 μM Met_3_SnCl. All points in scatter plots (time vs current) indicate events of closing or opening (of one, two, three or four SV channels). Current records have been processed and each closing-opening event in the sequence was assigned to its duration and average current amplitude. In the presence of 100 μM trimethyltin, the number of opening events was significantly reduced, what is evidenced particularly in histograms of opening times (Fig 5C and 5D). Histogram pertaining to the effect of trimethyltin covers a lower number of channel openings (Fig 5D), however it should be noted that its maximum is not significantly shifted compared to the control (Fig 5C). Both histograms, fitted with a lognormal distribution function, displayed similar peak positions (2.32 ms, σ = 0.97 in the control and 2.46 ms, σ = 1.18 in the presence of 100 μM trimethyltin). As presented in Fig 6, activity of single SV channels and the value of macroscopic currents obtained from measurements in the whole-vacuole configuration depended on trimethyltin concentration. The open probability (calculated as the total opening time normalized to the total time of record and the observed number of active channels in the patch) decreased with trimethyltin concentration in the incubation medium (Fig 6). For instance, at a concentration of 100 μM the open probability was reduced several times compared to the control. Interestingly, trimethyltin present in the incubation medium practically did not change the current-voltage characteristics of microscopic SV currents. Conductivity of single SV channels (slope of the current-voltage function) attained 65.1 ± 9.6 pS in the control and 62.0 ± 11.5 pS in the presence of 100 μM trimethyltin, suggesting that Met_3_SnCl did not affect the unitary conductance of channels.

## Discussion

Organotin compounds, as biologically active substances interacting with the cell membrane and organelle membranes, have a direct and indirect effect on ion channel proteins [15, 40, 41]. They may also influence the lipid phase of membranes and cause changes in their composition and liquidity, resulting in non-specific ion flow [15, 42, 43]. Trimethyltin chloride (Met_3_SnCl), studied in this work, is well known mainly as a potent neurotoxin. It triggers many neurodegenerative processes, including loss of neurons [44–46], as well as other degenerative membrane processes [47]. Trimethyltin has been also shown to decrease intracellular pH, open K^+^ channels and directly inhibit the activity of H^+^/K^+^-ATPase in renal intercalated cells [44]. It should be also added that in HeLa cells Met_3_SnCl disrupted cytosolic calcium homeostasis and produced elevated calcium transients [48, 49]. While on molecular level the toxic effect of organometals occurs at relatively low concentrations (10^−7^ to 10^−6^ M), the toxicity on cellular level manifested by changes in mechanical and physicochemical properties of cell walls and membranes appears at much higher concentrations of tin [50, 51]. Red beet vacuoles serve as a model for patch-clamp experiments which are crucial for understanding vacuole-related functions of plant cells. An important function of the vacuolar membrane (tonoplast) is the cytoplasm-vacuole transport of metabolites and ions [23, 52, 53], including the particularly important Ca^2+^ ions, involved in intracellular signalling [21, 54, 55] as well as sequestration of xenobiotics and heavy metals [56]. The last-mentioned are generally harmful substances, inducing free-radical processes and destruction of cell structure [56]. One of the most frequently examined transport systems across the tonoplast is the slow-activating vacuolar (SV) channel. Although SV channels have been well described (reviewed in [22, 23]), their physiological function *in vivo* remains poorly understood. So far, it has been well established that *in vitro* SV channels are activated by positive voltages and micromolar cytosolic Ca^2+^ concentrations [19]. Both SV and FV channels play an important function in the maintenance of potassium homeostasis, as by operating K^+^-sensing valves they control K^+^ distribution between vacuole and cytosol (recently reviewed in [57]).

The herein studied trimethyltin chloride irreversibly suppressed the macroscopic outward current in the tonoplast of *Beta vulgaris* taproots (Figs 1 and 2). Significant loss of macroscopic SV current occurred after several minutes of vacuole incubation in a medium containing trimethyltin. This observation may indicate either that Met_3_SnCl acts directly as a channel blocker or that it indirectly alters the kinetics of transition between the closed and open state of channel. Analysis of the kinetics of relaxation of macroscopic currents may be a source of information on channel gating. For voltage-dependent ion channels, at certain voltage the current reflects the distribution of the number of open channels to the total number of channels in the population. Stepping the membrane voltage (according to the measurement protocol) enforces relaxation of current among the various steady-state currents.

**Fig 7:**
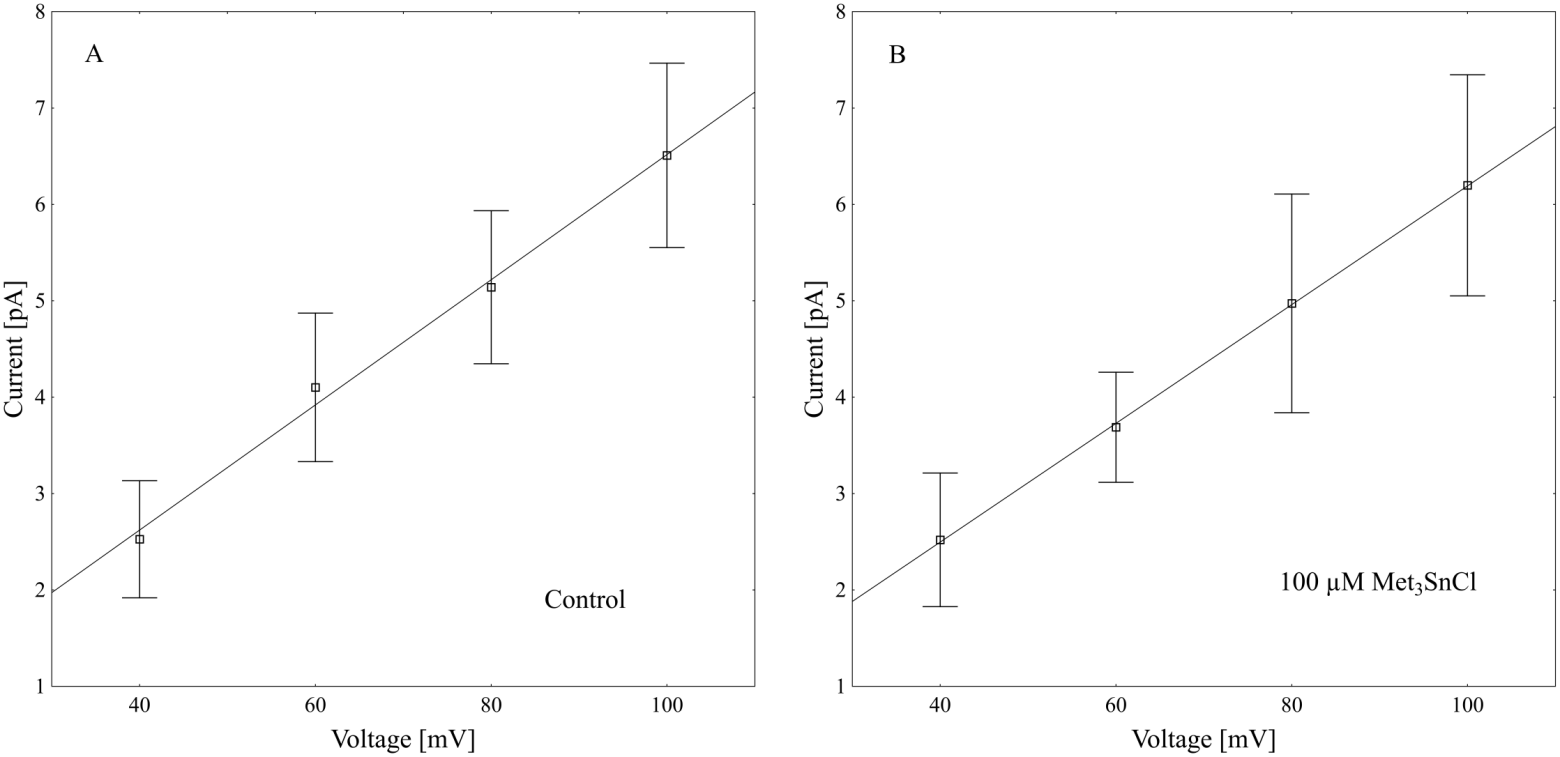
Current-voltage characteristics for microscopic SV current in control bath (A) and in the presence of 100 μM Met_3_SnCl (B). Points represent means (± SE, SD; n ≥ 4 independent experiments) for events of “open 1” recorded at selected voltages. As can be seen, Met_3_SnCl practically did not change the slope of simple linear regression, expressing the single channel conductivity (65.1 ± 9.6 pS in the control and 62.0 ± 11.5 pS in the presence of the organotin).

The time constant, τ, calculated by fitting macroscopic SV currents with a monoexponential function, showed that trimethyltin present in medium caused slowing of the macroscopic currents (ca. twofold) as compared to the control (Fig 3). Suppressed macroscopic SV current combined with slowed-down relaxation reflect the lower open probability of channels in the presence of organotin (Fig 4). Scatter plot of opening-closing events in the control and in the presence of trimethyltin chloride (Fig 5) shows a much lower number of openings after incubation in a medium containing the organometal. Trimethyltin changes the kinetics of the channel, however practically does not alter its electrical conductivity (Fig 7), what may suggest an indirect effect of Met_3_SnCl on the channel. The mechanism of SV channel modulation by trimethyltin chloride seems to be explained by its lipophilicity, especially that in the pH of the incubation medium used in the experiments (pH = 7.5) the non-dissociated (more lipophilic) form of the compound was dominant [58–60]. Experiments carried out on erythrocyte membranes, serving as model biological membranes, and on model lipid membranes showed that organic compounds of heavy metals, including tin, indeed affect membranes primarily due to their lipophilic properties [13, 15–17, 51, 61–63]. Such features of organotin compounds cause them to concentrate in the lipid phase of membranes and consequently disturb the interaction between lipids and proteins and therefore indirectly diminish SV channel activity. Similar results were obtained in our previous experiments, performed with the same plant material as here and concerning the effect of an organolead compound (trimethyllead chloride) on SV channels activity [37]. Both trimethyllead chloride and trimethyltin chloride significantly reduced the activity of SV channels, however causing only a small effect on their unitary conductance. This in turn may suggest that the Met_3_SnCl binding site, similarly to Met_3_PbCl [37], is located outside the channel selectivity filter. Interestingly, binding of the organotin compound near the selectivity filter of the ion channel of F-ATP synthase was also previously proposed by von Ballmoos et al. [64]. In agreement with their hypothesis hydrophobic organotin compounds accumulate within the membrane and easily penetrate into the entrance of the channel were they interact with a site near the selectivity filter disabling incoming ions to shed their hydration shell. Such scenario would be also possible in the case of our experiments on SV channels with Met_3_SnCl.

In conclusion, results presented here suggest that the suppression of SV currents by trimethyltin chloride arises probably from its hydrophobic properties allowing this compound to translocate near the selectivity filter of the channel.
